# 

**Published:** 1880-06-20

**Authors:** 


☞All Subscribers in arrears will please remit their subscription.

☞See card of C. R. Upson, M.D., Atlanta, Ga.

☞*Spectacles and Eye Glasses.*—Read the advertisement of S. Solomonson, jeweller and optician. Mr. Solomonson is a reliable gentleman, safe and moderate in his charges, and a good business man.

*Lactopeptine.*—We are in receipt of a beautiful sample of this valuable preparation from the Pharmacal Association of New York, for which our thanks are returned.

See their new advertisement in this journal.

*The Southern Medical College.*—This new and rising Institution will commence its second session, October 13th, 1880. The course of instruction in this school will be very full and thorough. The outlook is very encouraging for the future, and the founders are determined that it shall be second to no institution in the country.

---

*Annual Meeting of the American Association of Medical Colleges.*—This Association held its annual meeting in this city, in the afternoon of May 31st. Its principal work was the unanimous passage of a resolution recommending the adoption of three full courses in seperate years, as essential to obtaining a medical degree. It was also resolved that copies of the resolution be sent to the Association of Medical Editors, with a request for their co-operation in bringing about the three term course.—*The Medical Record.*

### *RECEIPTED.*

[Receipts not acknowledged privately are entered here.]

1880—Drs. E. L. McGehee, J. C. Webb, McCrady, H. E. Hurst, L. N. Hyten, J. B. Barwick, C. C. Jones, A. L. Barry, L. J. Sherrell, J. W. Hoff, J. A. Ashford, A. G. Smythe, F. T. Thorpe, E. H. Wright, T. E. Morris, F. C. Davis, W. J. Rogers, C. D. Tatman, J. J. Purcell, A. Harris, J. M. Rearden, J. L. Hamilton, E. R. Young, P. J. Parker, J. F. Brooks, W. K. Chambers, Z. T. Young, F. M. Fitzhugh, R. L. Hunter, J. E. Martin, H. E. Archer, S. Y. T. Carter, W. H. Boykin, H. Pinson, T. B. Meacham, E. A. Speed, P. A. Wilhite, J. L. Phelps, '79, J. M. Boring, '79.

